# Bidirectional Associations Between Longitudinal Change In Cognition And Instrumental Activities Of Daily Living

**DOI:** 10.1093/geroni/igaf122.2898

**Published:** 2025-12-31

**Authors:** Sydney Schaefer, Marie Ernsth-Bravell, Deborah Finkel

**Affiliations:** Arizona State University, Tempe, Arizona, United States; Jönköping University, Jönköping, Jonkopings Lan, Sweden; University of Southern California, Los Angeles, California, United States

## Abstract

Changes in daily function and cognition are two of the primary indicators of the aging process. However, how these two domains decline with respect to each other remains unclear. Recent work has suggested accelerated declines in fine motor function in instrumental activities of daily living (IADLs) precede the onset of dementia in older adults. However, whether early changes in IADLs contribute to subsequent changes in cognition remains unclear. Random intercept cross lag panel models (RI-CLPM) allow us to examine the temporal dynamics of the relationships between two variables that change over time and test hypotheses about direction of influence. This study applied age-based RI-CLPM to longitudinal changes in performance on a timed fine motor IADL test (which included five subtests, e.g., putting coins into a slot) and changes in the Mini-Mental Status Exam (MMSE). Up to 20 years of follow-up were available for 1,462 participants (aged 62-88 years at baseline) from three longitudinal Swedish datasets of older adults (OCTO-Twin, GENDER, SATSA). Model comparisons indicated a unidirectional relationship when participants were in their 60s and 70s: IADL time contributed to subsequent MMSE scores, but MMSE scores did not contribute to subsequent IADL time. For participants in their 80s, the relationship was bi-directional: both variables contributed to subsequent performance on the other variable. These data not only give insights into the temporal dynamics of decline in IADLs and cognition, but also provide proof-of-concept that tests involving motor performance, particularly when evaluated in ecologically-valid contexts, can be used for monitoring progression towards dementia.

